# Development and Temporal Validation of an Electronic Medical Record-Based Insomnia Prediction Model Using Data from a Statewide Health Information Exchange

**DOI:** 10.3390/jcm12093286

**Published:** 2023-05-05

**Authors:** Emma Holler, Farid Chekani, Jizhou Ai, Weilin Meng, Rezaul Karim Khandker, Zina Ben Miled, Arthur Owora, Paul Dexter, Noll Campbell, Craig Solid, Malaz Boustani

**Affiliations:** 1 Department of Epidemiology and Biostatistics, Indiana University Bloomington School of Public Health, Bloomington, IN 47405, USA; 2 Merck & Co., Inc., Rahway, NJ 07033, USA; 3 Department of Electrical and Computer Engineering, Indiana University-Purdue University Indianapolis, Indianapolis, IN 46202, USA; 4 Regenstrief Institute, Indianapolis, IN 46202, USA; 5 Department of Internal Medicine, Indiana University School of Medicine, Indianapolis, IN 46202, USA; 6 College of Pharmacy and Health Sciences, Purdue University, West Lafayette, IN 47907, USA; 7 Solid Research Group, LLC, Saint Paul, MN 55104, USA

**Keywords:** insomnia, sleep, machine learning, electronic medical records, temporal validation

## Abstract

This study aimed to develop and temporally validate an electronic medical record (EMR)-based insomnia prediction model. In this nested case-control study, we analyzed EMR data from 2011–2018 obtained from a statewide health information exchange. The study sample included 19,843 insomnia cases and 19,843 controls matched by age, sex, and race. Models using different ML techniques were trained to predict insomnia using demographics, diagnosis, and medication order data from two surveillance periods: −1 to −365 days and −180 to −365 days before the first documentation of insomnia. Separate models were also trained with patient data from three time periods (2011–2013, 2011–2015, and 2011–2017). After selecting the best model, predictive performance was evaluated on holdout patients as well as patients from subsequent years to assess the temporal validity of the models. An extreme gradient boosting (XGBoost) model outperformed all other classifiers. XGboost models trained on 2011–2017 data from −1 to −365 and −180 to −365 days before index had AUCs of 0.80 (SD 0.005) and 0.70 (SD 0.006), respectively, on the holdout set. On patients with data from subsequent years, a drop of at most 4% in AUC is observed for all models, even when there is a five-year difference between the collection period of the training and the temporal validation data. The proposed EMR-based prediction models can be used to identify insomnia up to six months before clinical detection. These models may provide an inexpensive, scalable, and longitudinally viable method to screen for individuals at high risk of insomnia.

## 1. Introduction

Insomnia is a common sleep disorder, with 30 to 40% of American adults experiencing symptoms in a given year [1] and as many as 50% of older adults reporting difficulty with sleep [2]. Insomnia etiologies are numerous and can be linked to biological, psychological, and social factors [3]. The risk of insomnia increases with age and is more common within certain populations, such as those with depression, anxiety, or traumatic brain injury [1]. The consequences of insomnia are significant. Insomnia is associated with diminished quality of life, impaired cognitive, physical, and occupational functioning, and an increased risk of mood disorders [4,5,6,7]. Among the elderly, insomnia has also been linked to falls, dementia, and institutionalization [8,9]. Annual insomnia-related costs are estimated to be 100 billion USD when considering both medical and indirect expenses (e.g., loss of productivity) [5,10].

Several effective insomnia treatments exist. Medications commonly used for insomnia include benzodiazepines, orexin antagonists, sedative-hypnotics, barbiturates, and melatonin agonists, although other classes of medications are sometimes used off-label [11,12]. While effective, pharmacotherapy is typically recommended for short-term use since there is the potential for adverse effects, dependency, and tolerance over time. Cognitive behavioral therapy for insomnia (CBT-I), an adaptation of traditional CBT designed to address sleep difficulties, is typically recommended as the first-line treatment option due to its superior safety profile [13]. However, CBT-I is often less accessible to patients than pharmacotherapy and requires sustained patient engagement for benefits to be realized [14].

Despite its prevalence and the widespread availability of effective therapies, insomnia often remains undetected and untreated; an estimated 70% of patients with insomnia report never discussing sleep with their doctor, and only 5% report scheduling a healthcare visit specifically to discuss their sleep problems [15]. Given the substantial burden of insomnia and the tendency of patients to not seek care, there is a significant opportunity to improve quality of life and reduce costs through better recognition and diagnosis. Identifying patients suffering from insomnia who have not yet received a formal diagnosis from a physician could enable early intervention, potentially reducing disease burden and healthcare utilization.

Several machine learning models have been developed to diagnose and screen for insomnia. These models used primary data from polysomnograms, magnetic resonance imaging, or electroencephalograms and achieved high accuracy [16,17,18,19]. Additionally, one study (Huang 2023) developed machine learning models to identify risk factors for patient-reported insomnia using the National Health and Nutrition Examination Survey (NHANES) data and achieved an AUC of 0.87 with an extreme gradient boosting (XGBoost) framework [20]. However, as these data sources are collected from surveys and expensive, non-routine tests, they are not available at scale for all patients. The widespread availability of electronic medical record (EMR) data offers an opportunity to develop inexpensive, targeted, and scalable processes to identify patients at risk for insomnia using only routine care data collected over a short surveillance period (i.e., 6 months to a year). Despite this potential, very few studies have explored leveraging routine care data from EMR for insomnia prediction; to date, only a single study has been published that developed an EMR-based insomnia prediction model. Kartoun et al. (2018) created an algorithm to identify patients with physician-documented insomnia using structured (i.e., demographics, ICD codes, and prescriptions) and unstructured (medical notes) EMR data [21]. The model achieved an AUC of 0.78 when relying on structured data alone, which increased to 0.83 when augmented with unstructured data from medical notes. The aim of this earlier study was to facilitate the identification of a large insomnia cohort for research purposes. It was limited to two hospitals and only included patients at risk of metabolic syndrome, making it unclear whether the model would generalize to the general adult population. Furthermore, the model was not temporally validated, so its longitudinal viability is unknown. In the present study, we aimed to develop and temporally validate a model that can identify insomnia in the general adult population using multi-institutional EMR data. Our secondary aim was to identify EMR-based predictors of insomnia risk.

## 2. Materials and Methods

This study was approved by the Indiana University Institutional Review Board (#11732) and adheres to the reporting standards described in the Transparent Reporting of Individual Prognosis or Diagnosis (TRIPOD) guidelines [22].

### 2.1. Cohort Selection

Study data were obtained from the Indiana Network for Patient Care (INPC). The INPC is one of the largest health information exchanges in the United States and includes EMR data for over 18 million patients across multiple healthcare systems [23]. This was a retrospective nested case-control study where cases were matched to controls by age within one year of the date of birth, sex, and race. Eligible cases were adults (≥18 years) with new insomnia between 1 January 2011 and 31 December 2018, defined as:

- a new insomnia diagnosis code (international classification of diseases, 9th or 10th clinical modification) or
- a new order for any of the following insomnia-specific medications: zolpidem, suvorexant, butabarbital, quazepam, estazolam, flurazepam, triazolam, tasimelteon, eszopiclone, temazepam, ramelteon, secobarbital, zaleplon, chloral hydrate, and melatonin. Antidepressants (e.g., doxepin, trazodone, and mirtazapine) and low-dose antipsychotics (e.g., quetiapine and olanzapine) with hypnotic properties were excluded from the list of medications used to identify insomnia cases because these off-label treatments may not be specific to insomnia.

Patients with insomnia diagnosis codes or medications before 2011 were considered to have prevalent insomnia and were thus ineligible. All patients were required to have at least one encounter with the INPC per year from 2010 to 2018 and have at least one diagnosis and medication record available during that time. Patients missing age, sex, or race information were excluded.

Patients without an insomnia diagnosis or medication were eligible to be selected as controls. Controls were matched to insomnia cases on a 1:1 basis by sex, race, and age within one year of the date of birth. If there was more than one potential control for a given case, a control was selected randomly from the pool of potential matches. In these cases, the index date was defined as the first date of an insomnia diagnosis code or medication. For controls, the index date was defined as the encounter date occurring closest to the matched case’s index date. EMR data from 2010 to 2018 was extracted to ensure that all patients had at least one year of pre-index data. Pre-index demographics, diagnosis, and medication data were processed to develop relevant exposure variables.

### 2.2. Socio-Demographic Variables

Demographic variables included age at index date, sex, and patient-reported race (categorized as White, Black, Other, and Unknown for analysis purposes). A higher cardinality for race is desirable. Unfortunately, since the data were collected from multiple health care institutions with varying patient race distributions and reporting requirements, there was a trade-off between a higher cardinality for the race variable and the number of patients included in the study. In addition to the above-listed demographic variables, we also considered insurance type, defined as the insurance reported at the visit closest to the index date and categorized as government (Medicare/Medicaid), commercial, self-pay, or other/unknown. The insurance type is a surrogate for the socioeconomic status of the patient, which has been previously reported to influence the risk of insomnia [24].

### 2.3. Diagnosis Variables

ICD-9-CM/ICD-10-CM diagnosis codes were used to define the 31 disease group variables (binary) that comprise the Elixhauser Comorbidity Index [25,26,27]. Elixhauser mortality scores were also calculated using Van Walravan weights [27]. To represent comorbidity burden, we also derived a new composite variable as the sum of the number of unique (at the 3-digit level) ICD-9-CM/ICD-10-CM codes a patient had during the pre-index period of interest. In total, 33 diagnosis variables were derived.

### 2.4. Medication Variables

Medication orders were extracted for all patients. Since these orders originated from multiple healthcare institutions, a unified mapping of medication names to a drug taxonomy was unavailable. Instead, each medication was mapped to the Anatomical Therapeutic Chemical (ATC) classification codes [28]. The ATC drug classification system is hierarchical with multiple sub-levels. For this study, the first-level sub-groups of all 14 main ATC groups were included (e.g., A01: stomatological preparations, A02: drugs for acid-related disorders, etc.). For each patient, the number of medication orders associated with a given ATC sub-group was calculated over the surveillance period of interest. This processing led to 98 medication variables.

### 2.5. Model Development and Evaluation

In total, 135 variables were derived from demographics, diagnosis, and medication order data. One-hot encoding was performed on all categorical variables, and all continuous variables were standardized. Initially, we explored five candidate machine learning models to predict insomnia: logistic regression, support vector machine with the radial basis function kernel, random forest, extreme gradient boosting (XGBoost), and a multilayer neural network. For model selection, data from 2011–2017 (based on patient index date) were randomly split into a training set (80%) and a holdout set (20%) while maintaining 1:1 case/control to avoid class imbalance. Each candidate model was evaluated by calculating the area under the receiver operating characteristic curve (AUC) on the holdout set. XGBoost had the highest AUC out of the five candidates and was therefore selected as the model of choice. A voting ensemble architecture consisting of the top three performing models was also considered but demonstrated no significant (<1%) improvement over the XGBoost model alone.

After deciding on XGBoost, the data were split into three subsets based on patient index date (2011–2013, *n* = 15,008; 2011–2015, *n* = 25,372; and 2011–2017, *n* = 34,850). The purpose of subsetting the data was to create models with different time periods and observe their performance over subsequent time periods. For each of these subsets, we created separate XGBoost models using data derived from the following surveillance periods before the index date: (1) −1 to −365 days before index, and (2) −180 to −365 days before index, thereby allowing for an understanding of how the surveillance period impacts the models’ ability to detect patients at risk for insomnia. Before training, each model’s data were randomly split into a training set (80%) and a holdout set (20%) while again maintaining 1:1 case/control to avoid class imbalance. Hyperparameter tuning was performed using a grid search approach with 5-fold cross-validation. We evaluated the predictive performance of each model on its corresponding holdout set by creating 1000 bootstrapped samples with replacement, calculating the AUC for each sample, and averaging the results. SHapley Additive exPlanation (SHAP) [29] was used to determine which features had the strongest influence on model predictions. Calibration was assessed using calibration curves.

### 2.6. Temporal Validation

**Figure 1:**
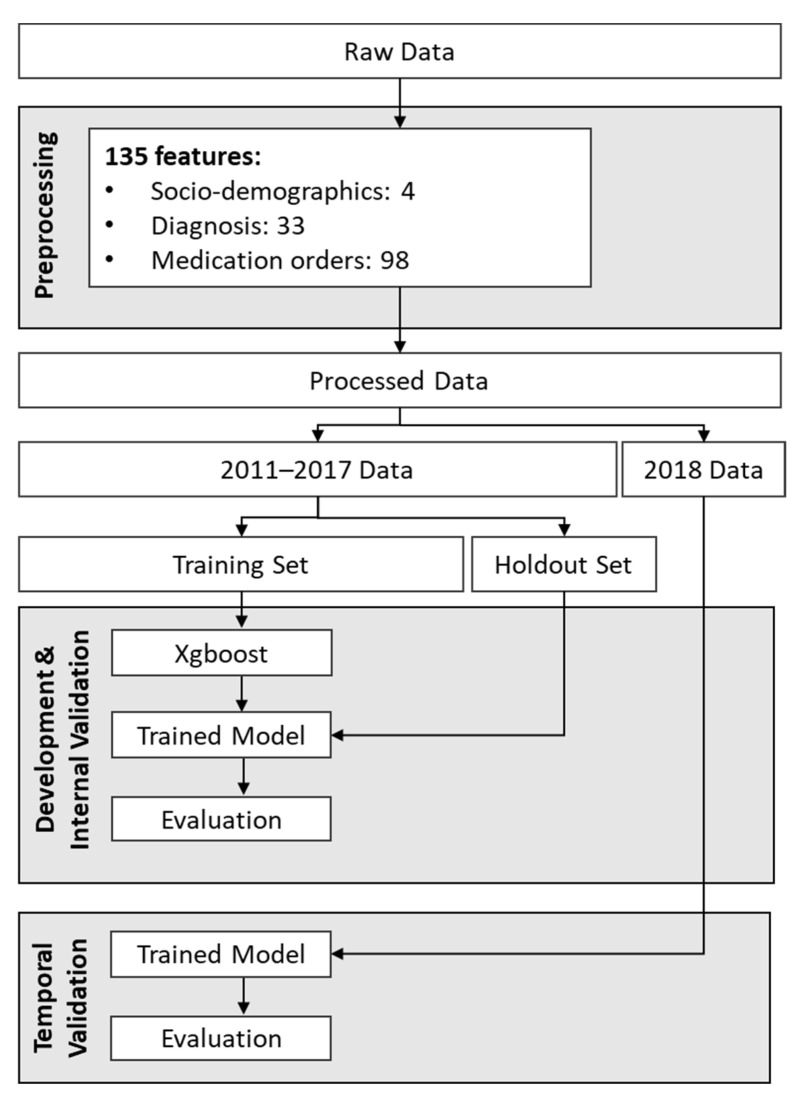
Workflow for development and temporal validation of the model developed using data from 2011 to 2017.

Temporal validation was performed to examine how the models’ performances would change over time. To achieve this, we evaluated each model’s performance on future data, one year at a time, up to 2018. Relatively stable performance over time would indicate that the model is low-maintenance and could potentially be used in production within a healthcare system for several years without requiring retraining. Figure 1 shows the workflow used for model development, internal validation, and temporal validation for the insomnia prediction model trained on patient data from 2011–2017.

## 3. Results

### 3.1. Study Sample

**Figure 2:**
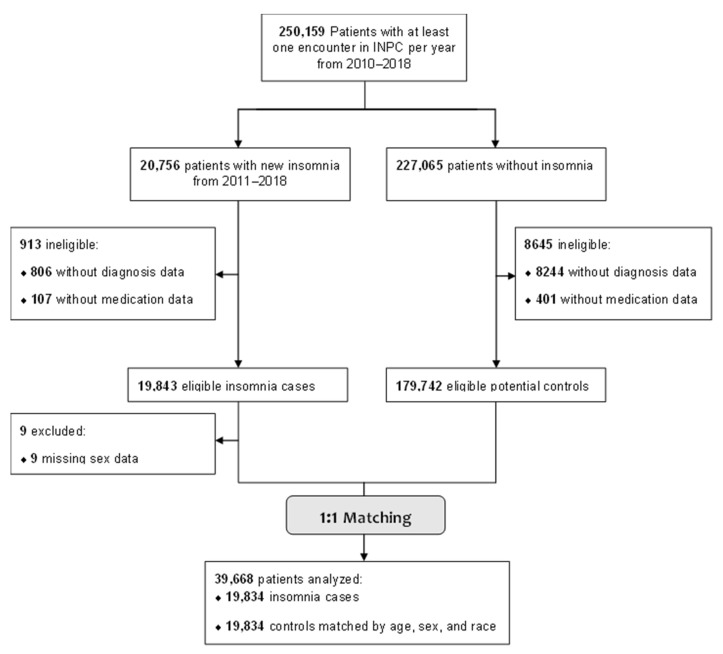
Flow Diagram for Patient Inclusion in the Insomnia Risk Prediction Model.

There were 250,159 adult patients with at least one encounter per year during the study period, 19,834 of whom had a new insomnia ICD-9-CM/ICD-10-CM code or insomnia medication between 2011–2018. Of the identified insomnia cases, 913 were ineligible due to not having any medication or diagnosis data (potentially due to data entry errors), and nine were excluded for missing sex data. The final analysis sample included 39,668 patients (19,834 insomnia cases and 19,834 matched controls, Figure 2). Overall, the cohort was primarily female (70%), white (78%), and publicly insured (44%), with a median age of 59 years (Table 1). The five most common diagnoses in the study sample were hypertension (33%), diabetes (17.1%), hypothyroidism (11%), and chronic pulmonary disease (9%). Compared to controls, insomnia cases had a greater comorbidity burden (Table 2).

For most cases, the first instance of insomnia was defined by an order for an insomnia medication (59.4%) rather than an ICD-9-CM/ICD-10-CM code (40.6%). When considering the entire study period, 9543 (53.3%) cases had an ICD-9-CM/ICD-10-CM documented at some point, and 13,213 (63.5%) cases were prescribed an FDA-approved insomnia medication. Among those who received pharmacotherapy, the most frequently prescribed insomnia-specific medications were zolpidem (61.7%), temazepam (13.5%), triazolam (7.8%), and eszopiclone (7.6%).

### 3.2. Model Selection

The five candidate classifiers were developed and evaluated using data from 2011–2017 with a surveillance period of −1 to −365 days before the index. The training set consisted of 13,940 cases and 13,940 controls; the holdout set consisted of 3485 cases and 3485 controls. XGBoost produced the highest AUC (0.80) on the holdout set by a narrow margin compared to the neural network and random forest models, which both had AUCs of 0.79. The SVM and logistic regression models performed the worst, with an AUC of 0.74 and 0.68, respectively. A voting ensemble architecture combining the top three models was also evaluated. However, no significant (<1%) improvement was observed over the XGBoost model alone. Therefore, this latter model was retained for further analysis.

### 3.3. Prediction Models

The holdout set AUCs for models trained on data from 2011–2013 (*n* = 14,970), 2011–2015 (*n* = 25,286), and 2011–2017 (*n* = 34,850) were nearly identical, although models trained on fewer samples tended to have greater variability in performance (Table 3). The models trained on data with a surveillance period of −1 to −365 days before the index had AUCs of approximately 0.80, indicating good predictive performance. Models that used data closer to a patient’s index date performed better than those using data further from the index date; when the surveillance period was limited to −180 to −365 days before the index date, AUCs decreased (range 0.68–0.70, Table 3). The above two surveillance periods correspond to 1-day and 6-month prediction horizons prior to the onset of the disease. For clinical decision support, the earlier the risk prediction, the better. Unfortunately, extending the prediction horizon using a surveillance period of −730 to −365 days prior to the index date (i.e., a one-year prediction horizon) resulted in an inconclusive risk prediction model. We hypothesize that a surveillance period that is closer to the index date is needed for insomnia risk prediction because trigger factors related to medications and comorbid conditions are immediate rather than delayed. This is supported by the most important predictors of the models (Table 4). The number of orders for analgesic medications was the most important predictor for all models with a surveillance period of −1 to −365 days before index. In comparison, insurance was the most important predictor for the models with a surveillance period of −180 to −365 days. The models were well calibrated for both surveillance periods (Figures S1 and S2).

### 3.4. Temporal Validation

In general, AUCs were stable over time. For example, the −1 to −365 model trained on the fewest samples (2011–2013) had an AUC of 0.80 when making predictions using holdout patient data within the same period; see Table 3. For patient data one year (2014) and five years (2018) in the future, AUCs of 0.78 and 0.76 are obtained using the same model, respectively. These results indicate a minimal decrease in performance as the patient populations shift over time. Similar results were observed for the 2011–2015 and 2011–2017 models.

## 4. Discussion

Our results indicate that multivariable prediction models can successfully identify patients at high risk of insomnia using routine EMR data. In our experiments, an XGBoost model trained using a combination of demographics, diagnosis, and medication data captured −1 to −365 days before the index date achieved an average AUC of 0.80 over the holdout dataset and was well calibrated. Furthermore, temporal validation demonstrated that the model’s performance is stable when making predictions up to five years in the future, suggesting that it may require relatively little maintenance once deployed. EMR-based prediction models like those presented in this study have been successfully used to detect prodromal Alzheimer’s disease [30]. To our knowledge, this is the first study to develop and temporally validate an insomnia prediction model using multi-institutional EMR data.

Psychotropic medications (psychoanaleptics and psycholpetics) and analgesic medications were strong predictors of insomnia in the model. The fact that these medication groups correspond to conditions frequently associated with insomnia in the literature, including mood disorders, chronic pain, and gastroesophageal reflux disease, or are known to cause insomnia as a side effect (e.g., stimulants), supports these models being clinically explainable [31,32,33,34]. Insurance type was also a top feature, which may reflect an association between the ability to work and better health; while cases and controls were matched by age, significantly more cases had government insurance (Medicare or Medicaid) than controls (49% vs. 38%, respectively). Elixhauser mortality score was missing from the top five most important features. However, the number of comorbidities appeared frequently. This may suggest that the number of individual conditions matters more than aggregated mortality scores when predicting insomnia. One possible explanation is that individuals with a greater number of comorbidities experience greater psychological distress, which, in conjunction with their underlying illnesses, increases their risk of insomnia.

Our results indicate that the length and proximity of the surveillance period to the index date can significantly impact the accuracy of insomnia risk prediction when using EMR data. All performance metrics increased with surveillance duration and proximity, which was expected given that the −1 to −365 models had access to more recent information than the −180 to −365 models. The fact that the −1 to −365 models performed better suggests that the diagnoses and medications that best predict insomnia occur closer to the index date and may not be consistently captured when considering a distant surveillance period. This lack of documentation was reflected in the lists of important features; for example, four of the five top features in the −1 to −365 models were clinical variables (medication orders and number of comorbidities). In contrast, the model with a surveillance period ranging from −180 to −365 days before the index had top features such as age and insurance type, which are independent of the surveillance period and clinically agnostic.

As previously mentioned, the AUCs of the −1 to −365 models all indicate good discriminative ability. However, it is also important to determine whether the model’s performance is stable over time—that is, whether it will need frequent retraining after being deployed in a healthcare system. Our models’ performance remained stable when making predictions on data up to five years in the future, suggesting they are longitudinally viable.

While several studies have explored insomnia prediction using primary data, published research using EMR data is limited. The model proposed by Kartoun et al. (2018) mentioned earlier achieved an AUC of 0.78 when using structured EMR data, which is similar to the present study [16]. However, compared to the present study, this earlier model did not consider patients taking insomnia-specific medications without a documented diagnosis. Moreover, over 75% of patients in the study had at least ten years of EMR records. This extended surveillance period should be compared to the 6- and 12-month surveillance periods reported in the present study.

In summary, this study shows that a model that depends on a limited set of routinely collected variables and a one-year surveillance period can deliver accurate insomnia risk prediction. This finding is important because, even though insomnia is common, patients are often reluctant to discuss sleep issues with their physicians and may delay seeking help with their insomnia until it becomes severe [15]. The models presented in this study offer an inexpensive and scalable method to screen for individuals at high risk of insomnia who could benefit from additional, targeted assessment by a healthcare professional. Because it uses routine EMR data, the model can be readily integrated into existing EMR systems to support clinical decision-making and early treatment initiatives.

Our study has several strengths. First, the data were sourced from a statewide health information exchange, resulting in a large and diverse study sample. Second, we compared models with different surveillance periods to examine how surveillance length affects predictive performance. We also used both ICD codes and insomnia-specific medications to improve insomnia case identification and avoid potential misclassification. Finally, we temporally validated the models to determine their stability over time, an important practical consideration as a temporally sensitive model would require frequent retraining and updates.

This study also has important limitations. While the INPC receives information from most major healthcare systems in Indiana, encounters at non-INPC facilities may not have been captured. Therefore, INPC may not be representative of independent healthcare centers, so our results may not generalize to those populations. Additionally, routine care EMR data may not include all relevant information for insomnia prognostication. Off-label insomnia medications were not considered because they are indicated for the treatment of depression, pain, psychosis, and other medical conditions and would require a review of treatment causes from medical notes to ascertain their relevance to insomnia. As such, insomnia patients without an insomnia ICD code who were treated with off-label medications would have been missed. That said, off-label insomnia medication and the use of medical notes as an additional source of data are being considered for future work.

Requirements for matching cases and controls enforced in the study design were intended to limit biases related to age, sex, and race. However, biases resulting from other criteria, such as off-label medications, insurance types, and health service providers, were not considered.

Nonetheless, with a surveillance period of 1 year, the proposed model achieved an AUC of 0.8 and was temporally validated over five years. As such, we believe it is suitable as a pre-screening method. The model can be applied frequently to all patients without any additional burden. The medical charts of patients identified as at risk by this method should then be reviewed by a health care provider. In addition, we believe that the present study demonstrates the potential of an algorithmic approach to insomnia prognostication and provides a baseline for future enhancements, including the use of medical notes, deep-learning models, and a larger cohort of patients.

## 5. Conclusions

Our findings suggest that EMR-based prediction models may provide an inexpensive, scalable, and longitudinally viable method to identify individuals at risk of insomnia. Our models demonstrated clinically useful discriminative performance in the general adult population and remained stable over time. Clinical application of the proposed models as an automated screening tool could facilitate targeted, physician-led assessments and subsequent early intervention, thereby potentially reducing insomnia-related healthcare burden and improving quality of life for patients.

## Figures and Tables

**Table 1:** Demographic Characteristics for Cases and Controls, Entire Cohort (2011–2018).

Variables	Control (*n* = 19,834)	Case (*n* = 19,834)	Total (*n* = 39,668)
Age *, years, median (IQR)	59 (48–70)	59 (48–70)	59 (48–70)
Female	13,897 (70%)	13,897 (70%)	27,794 (70%)
Race group, *n* (%)			
Black	1749 (8.8%)	1749 (8.8%)	3498 (8.8%)
White	15,553 (78%)	15,553 (78%)	31,106 (78%)
Unknown	2197 (11%)	2197 (11%)	4394 (11%)
Other	335 (1.7%)	335 (1.7%)	670 (1.7%)
Insurance type, *n* (%)			
Government	7630 (38%)	9791 (49%)	17,421 (44%)
Private	9321 (47%)	6255 (32%)	15,576 (39%)
Self-pay	598 (3.0%)	888 (4.5%)	1486 (3.7%)
Other/Unknown	2285 (12%)	2900 (15%)	5185 (13%)

* At index date. Controls were matched to insomnia cases by age within 1 year, sex, and race.

**Table 2:** Clinical Characteristics for Cases and Controls, Entire Cohort (2011–2018).

Variables	Control (*n* = 19,834)	Case (*n* = 19,834)	Total (*n* = 39,668)
Elixhauser Comorbidity Score, median (IQR)	0 (0–0)	0 (0–5)	0 (0–3)
Number of Comorbidities, median (IQR)	3 (0–8)	8 (2–15)	5 (1–11)
Elixhauser Comorbidity Group, *n* (%)			
Congestive heart failure	411 (2.1%)	1066 (5.4%)	1477 (3.7%)
Cardiac arrhythmias	1096 (5.5%)	2050 (10%)	3146 (7.9%)
Valvular disease	437 (2.2%)	685 (3.5%)	1122 (2.8%)
Pulmonary circulation disorders	146 (0.7%)	370 (1.9%)	516 (1.3%)
Peripheral vascular disorders	486 (2.5%)	947 (4.8%)	1433 (3.6%)
Hypertension, uncomplicated	4766 (24%)	7121 (36%)	11,887 (30%)
Hypertension, complicated	392 (2.0%)	795 (4.0%)	1187 (3.0%)
Paralysis	49 (0.2%)	158 (0.8%)	207 (0.5%)
Other neurological disorders	369 (1.9%)	969 (4.9%)	1338 (3.4%)
Chronic pulmonary disease	1108 (5.6%)	2474 (12%)	3582 (9.0%)
Diabetes, uncomplicated	1945 (9.8%)	3099 (16%)	5044 (13%)
Diabetes, complicated	553 (2.8%)	1076 (5.4%)	1629 (4.1%)
Hypothyroidism	1821 (9.2%)	2602 (13%)	4423 (11%)
Renal failure	699 (3.5%)	1219 (6.1%)	1918 (4.8%)
Liver disease	336 (1.7%)	702 (3.5%)	1038 (2.6%)
Peptic ulcer disease	27 (0.1%)	85 (0.4%)	112 (0.3%)
AIDS/HIV	68 (0.3%)	130 (0.7%)	198 (0.5%)
Lymphoma	207 (1.0%)	429 (2.2%)	636 (1.6%)
Metastatic cancer	98 (0.5%)	238 (1.2%)	336 (0.8%)
Solid tumor without metastasis	1272 (6.4%)	1800 (9.1%)	3072 (7.7%)
RA/collagen vascular disease	473 (2.4%)	930 (4.7%)	1403 (3.5%)
Coagulopathy	190 (1.0%)	412 (2.1%)	602 (1.5%)
Obesity	715 (3.6%)	1756 (8.9%)	2471 (6.2%)
Weight loss	180 (0.9%)	523 (2.6%)	703 (1.8%)
Fluid and electrolyte disorders	655 (3.3%)	1709 (8.6%)	2364 (6.0%)
Blood loss anemia	53 (0.3%)	140 (0.7%)	193 (0.5%)
Deficiency anemia	719 (3.6%)	1405 (7.1%)	2124 (5.4%)
Alcohol abuse	67 (0.3%)	227 (1.1%)	294 (0.7%)
Drug abuse	74 (0.4%)	329 (1.7%)	403 (1.0%)
Psychoses	147 (0.7%)	611 (3.1%)	758 (1.9%)
Depression	696 (3.5%)	2521 (13%)	3217 (8.1%)

Note: Comorbidity status was ascertained using data from −1 to −365 days before the index date. AIDS = acquired immunodeficiency syndrome; HIV = human immunodeficiency virus; RA = rheumatoid arthritis.

**Table 3:** Evaluation and Temporal Validation of XGBoost Insomnia Prediction Models.

	Training Data and Surveillance Period					
	2011–2013 (*n* = 14,970)		2011–2015 (*n* = 25,286)		2011–2017 (*n* = 34,850)	
	−1 to −365	−180 to −365	−1 to −365	−180 to −365	−1 to −365	−180 to −365
Holdout * AUC	0.80 (0.008)	0.70 (0.009)	0.79 (0.006)	0.68 (0.008)	0.80 (0.005)	0.70 (0.006)
2014 AUC	0.78 (0.007)	0.68 (0.008)	--	--	--	--
2015 AUC	0.76 (0.006)	0.69 (0.007)	--	--	--	--
2016 AUC	0.77 (0.006)	0.69 (0.007)	0.79 (0.006)	0.74 (0.007)	--	--
2017 AUC	0.77 (0.007)	0.71 (0.008)	0.79 (0.007)	0.74 (0.008)	--	--
2018 AUC	0.76 (0.007)	0.68 (0.008)	0.77 (0.007)	0.71 (0.007)	0.77 (0.007)	0.72 (0.007)

* Holdout sets were derived from the same time period as the training data. The mean AUC (SD) obtained from 1000 bootstrapped samples with replacement. A 1:1 ratio of cases to controls was maintained across all data.

**Table 4:** Top five Most Important Features in XGBoost models’ predictions by surveillance period.

Data	Rank *	Surveillance Period	
		−1 to 365 Days	−180 to −365 Days
2011–2013 (holdout)	1	Analgesic medications	Private insurance
	2	Psychoanaleptic medications	Analgesic medications
	3	Private insurance	Psychoanaleptic medications
	4	Psycholeptic medications	Number of comorbidities
	5	Antiepileptic medications	Psycholeptic medications
2011–2015 (holdout)	1	Analgesic medications	Private insurance
	2	Psychoanaleptic medications	Analgesic medications
	3	Private insurance	Psychoanaleptic medications
	4	Psycholeptic medications	Age
	5	Number of comorbidities	Psycholeptic medications
2011–2017 (holdout)	1	Analgesic medications	Private insurance
	2	Psychoanaleptic medications	Analgesic medications
	3	Number of comorbidities	Psychoanaleptic medications
	4	Private insurance	Number of comorbidities
	5	Psycholeptic medications	Age

* Feature importance is measured using SHAP values.

## Data Availability

The data that support the findings of this study are available from the Regenstrief Institute, but restrictions apply to the availability of these data, which were used under a research agreement for the current study and are therefore not publicly available. Data are, however, available from the senior author (M.B.) upon reasonable request and with permission of the Regenstrief Institute.

## Supplementary Materials

The following supporting information can be downloaded at: https://www.mdpi.com/article/10.3390/jcm12093286/s1, Figure S1: Calibration curve for 2011–2017 XGBoost model on holdout data (−1 to −365 days surveillance period); Figure S2: Calibration curve for 2011–2017 XGBoost model on holdout data (−180 to −365 days surveillance period).
