# Supplementary material for: Development and Temporal Validation of an Electronic Medical Record-Based Insomnia Prediction Model Using Data from a Statewide Health Information Exchange
Source: J Clin Med. 2023 May 5;12(9):3286. doi: 10.3390/jcm12093286 (PMC10179562; doi:10.3390/jcm12093286)

Figure S1. Calibration curve for 2011–2017 XGBoost model on holdout data (-1 to -365 days surveillance period)

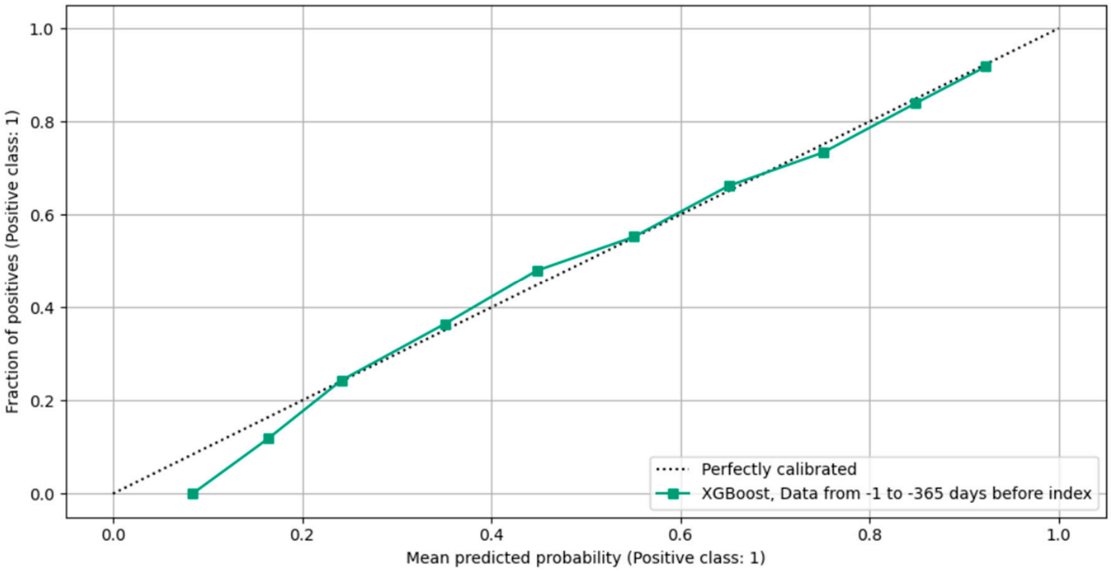

Figure S2. Calibration curve for 2011–2017 XGBoost model on holdout data (-180 to -365 days surveillance period)

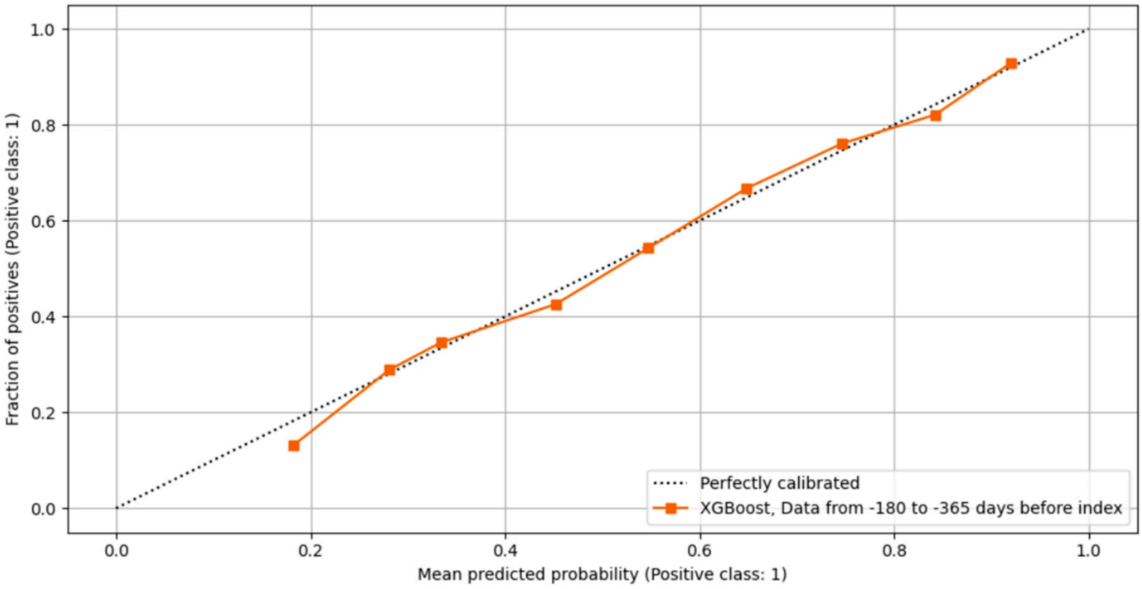

Supplement: Supplementary file 1 [file jcm-12-03286-s001.zip › jcm-2368242-supplementary.pdf]
